
JOURNAL INFORMATION
==============================
NLM Title Abbreviation: ScientificWorldJournal
Journal ID: ScientificWorldJournal
NLM Journal ID: 101131163
Journal ID: TSWJ

The Scientific World Journal

ISSN: 2356-6140
EISSN: 1537-744X
Publisher: Wiley

ARTICLE INFORMATION
==============================
PMCID: PMC6009707
PMID: 29973820
DOI: 10.1100/tsw.2002.46
Article ID: 857597
Article version: 1
Subjects: Short Report

Thermodynamic Molecular Switch in Sequence-Specific Hydrophobic Interactions

Chun Paul W. *
Department of Biochemistry and Molecular Biology, University of Florida College of Medicine, Box 100245, Gainesville, FL 32610-0245, USA
*Paul W. Chun: pwchun@biochem.med.ufl.edu
Publication date: 2002
Electronic publication date: 2002 Jun 27
Volume: 2
First page: 97
Last page: 98
Copyright: Copyright © 2002 Paul W. Chun.
Copyright year: 2002
License: This is an open access article distributed under the Creative Commons Attribution License, which permits unrestricted use, distribution, and reproduction in any medium, provided the original work is properly cited.
License URL: https://creativecommons.org/licenses/by/3.0/

==============================
Advances in Gene Technology: The Genome and Beyond –
Structural Biology for Medicine (Proceedings of the 2002 Miami
*Nature Biotechnology* Winter Symposium)
*TheScientificWorld* 2002, 2(S2), 97–98
ISSN 1532-2246; DOI 10.1100/tsw.2002.46

### THERMODYNAMIC MOLECULAR SWITCH IN SEQUENCE-SPECIFIC HYDROPHOBIC INTERACTIONS

Paul W. Chun
Department of Biochemistry and Molecular Biology, University of Florida College of Medicine,
Box 100245, Gainesville, FL 32610-0245
*pwchun@biochem.med.ufl.edu

**INTRODUCTION.** We have shown in our published work the existence of a thermodynamic switch in biological systems wherein a change of sign in $\Delta Cp^{o}(T)_{reaction}$ leads to a true negative minimum in the Gibbs free energy change of reaction, and hence, a maximum in the related $K_{eq}$. This presentation demonstrates the existence of a thermodynamic molecular switch in the pair-wise, sequence-specific hydrophobic interaction of Ile-Ile, Leu-Ile, Val-Leu, or Ala-Leu over the temperature range of 273–333 K, as reported by Nemethy and Scheraga[1] and analyzed by the Plank-Benzinger methodology[2–7].

**METHOD.** In order to analyze the thermodynamic processes operating in a pair-wise hydrophobic interaction such as isoleucine-isoleucine, it is necessary to extrapolate the thermodynamic parameters over a much broader temperature range. The enthalpy, entropy, and heat capacity terms are evaluated as partial derivatives of the Gibbs free energy function defined by Helmholtz-Kelvin expression[8]. The rationale for selecting the linear third-order polynomial function for $\Delta G^{o}(T) = \alpha + \beta T^{2} + \gamma T^{3}$ (macromolecular interaction) and $\Delta H(T) = \alpha + \beta T^{3} e^{\gamma T}$ (protein unfolding) are found in the fundamentals of relevant quantum theory[9].

**RESULTS.** Based on Chun's development of the Planck-Benzinger methodology, the change in inherent chemical bond energy at 0 K, $\Delta H^{o}(T_{0})$, is 3.0 kcal $mol^{-1}$ for Ile-Ile, 2.4 for Leu-Ile, 1.8 for Val-Leu, and 1.2 kcal $mol^{-1}$ for Ala-Leu. The value of $\Delta H^{o}(T_{0})$ decreases as the length of the hydrophobic side chain decreases. It is clear that the strength and stability of the hydrophobic interaction is determined by the packing density of the side chains, with Ala-Leu being the most stable. At $\langle T_{m} \rangle$, the thermal agitation energy, is about five times greater than $\Delta H^{o}(T_{0})$ in each case. Additionally, the thermal agitation energy for the same series, evaluated at $\langle T_{m} \rangle$, decreases in the same order, which is as the length of the side chain decreases. This pair-wise, sequence-specific hydrophobic interaction is highly similar in its thermodynamic behavior to that of other biological systems, except that the negative Gibbs free energy change minimum at $\langle T_{s} \rangle$ occurs at a considerably higher temperature, 355 K compared to about 300 K. The melting temperature, $\langle T_{m} \rangle$, is also high, 470 K compared to 343 K in a biological system.

**DISCUSSION**. The implication is that the negative Gibbs free energy minimum at a well-defined <$T_s$> has it origin in the hydrophobic interactions, which are highly dependent on details of molecular structure. We have shown in our unpublished work the existence of a thermodynamic molecular switch in the pair-wise hydrophobic interactions of 32 dipeptides. Indeed, all interacting biological systems that we have thus far examined using the Planck-Benzinger approach point to the universality of this thermodynamic molecular switch.

**REFERENCES**

1. Nemethy, G. and Scheraga, H.A. (1962) J. Phys. Chem. 66, 1773–1789.
2. Chun, P.W. (1998) Methods Enzymol. 295, 227–268.
3. Chun, P.W. (1999) Int. J. Quantum Chem. 75, 1027–1042.
4. Chun, P.W. (2000) Biophys. J. 78, 416–429.
5. Chun, P.W. (2000) Cell Biochem. Biophys. 33, 149–169.
6. Chun, P.W. (2000) Int. J. Quantum Chem. 80, 1181–1198.
7. Chun, P.W. (2001) Colloids Surfaces 181, 183–169.
8. Moelywn-Hughes, E.A. (1957) Physical Chemistry. Pergamon Press, New York. pp 90–103, 264–279, 831–837.
9. Planck, M. (1927) Vorlessungen uber Thermodynamics. 7th ed. Ogg, A., Transl. Treatise on Thermodynamics. Longmans, London, pp 164–182, 665–668.
